# Mucormycosis infection in hematopoietic stem cell transplant patients: A serious threat

**DOI:** 10.1016/j.amsu.2022.104353

**Published:** 2022-09-09

**Authors:** Syeda Lamiya Mir, Saboor Ahmed, Laraib Ghanghro, Priya, Govinda Khatri, Mohammad Mehedi Hasan

**Affiliations:** a Dow University of Health Sciences, Karachi, Pakistan; b Department of Biochemistry and Molecular Biology, Faculty of Life Science, Mawlana Bhashani Science and Technology University, Tangail, Bangladesh

Mucormycosis is an intrusive contagious disease caused by the Mucorales fungus. The infection spreads mostly by spore inhalation, skin wounds, catheters and needles, and contaminated food. Immunocompromised people in developed countries, such as those with hematological malignancies, organ transplants, and neutropenia, are at risk. Diabetics and those who have endured significant trauma are the most vulnerable to mucormycosis infection in impoverished nations [1].

It has the ability to migrate hematogenous from the site of infection to other body locations and generate thrombosis and tissue infarction, which is a feature of mucormycosis infection. In the battle against infection, phagocytes are an important host defense mechanism [2]. The primary site of infection varies according to the susceptible group, with the skin being the most common in the healthy population, the paranasal sinuses in diabetics, and the lungs in HSCT patients. Mucormycosis has become increasingly common in those who have had hematopoietic stem cell transplantation (HSCT) or solid organ transplantation (SOT). This is due to the immunosuppressive medication used during the HSCT procedure. Mucormycosis is more common in allogeneic HSCT patients than in autologous HSCT patients [3].

Mucormycosis has a significant morbidity and death incidence in those who have undergone hematopoietic stem-cell transplantation (HSCT). According to recent studies, the incidence of Mucormycosis in HSCT patients is predicted to be around 5% [3]. It is typically a late result of HSCT, with reported median incidence lengths ranging from 135 to 225 days. The disease-related mortality rate has been calculated to be 75%, with a median survival duration of fewer than 2 months [4]. Severe graft versus host disease (GVHD), a high steroid dosage, a history of CMV or respiratory viral infection, and increasing age are all risk factors for mucormycosis in HSCT patients [4]. According to recent research, diabetes, malnutrition, and voriconazole prophylaxis were all possible risk factors for mucormycosis in HSCT patients [5]. Although several cases of mucormycosis have been reported in voriconazole-treated HSCT patients, the particular function of the medication in mucormycosis prevalence remains unknown [6].

Notably, there are no biomarkers available to detect mucormycosis infection in HSCT patients. The galactomannan and -D-glucan assays for Aspergillus cannot identify antigen components of the Mucorales cell wall. To identify mucormycosis from more prevalent and antifungal-sensitive molds like Aspergillus, a direct investigation of fluids and cultures taken from sterile regions is essential [7]. The findings of direct inspection were positive in 79% of the 29 HSCT patients with recently proved or suspected mucormycosis and culture results were positive in 86% [8].

More than half of HSCT patients having mucormycosis exhibit lung infection, 10%–15% have central nervous system involvement, and 0%–40% have sinus involvement. The disseminated illness affects at least 10% of HSCT patients with mucormycosis [9,10]. Invasive fungal infection prevention and treatment may improve clinical outcomes. Effective therapeutic options include primary and secondary prevention, empirical antifungal medication, and guided treatment of existing fungal infections. Fungal infections have grown increasingly common in bone marrow transplant patients over time [11].

Enhanced immunological and metabolic aspects, such as the use of tapered dosages of steroids and immunosuppressive drugs when possible, and hyperglycemia control, should be regularly monitored in the therapy of mucormycosis in SOT and HSCT patients [7]. Given the rapid development rate of Mucorales and the fact that 84% of patients with lung mucormycosis get unsuccessful treatment at the time of diagnosis, it is reasonable to infer that the window of opportunity for effective medication is substantially narrower than that for aspergillosis [12].

Three critical techniques for addressing mucormycosis in hematological patients are (1) prompt initiation of effective antifungal medication, (2) substantial “early” surgical dissection of necrotic lesions, and (3) speedy control of underlying disease, when feasible [13]. Only two systemic antifungals with Mucorales activity are currently available: amphotericin B lipid formulation with triazole Posaconazole [14].

## Ethics statement

The present study includes printed and published information; therefore, formal ethical clearance was not applicable for this study.

## Sources of funding

NA.

## Ethical approval

NA.

## Consent

NA.

## Author contribution

All authors meet the inclusion criteria, and all authors read and approved the final version of the manuscript.

## Registration of research studies

Name of the registry: NA.

Unique Identifying number or registration ID: NA.

Hyperlink to your specific registration (must be publicly accessible and will be checked): NA.

## Guarantor

Mohammad Mehedi HasanDepartment of Biochemistry and Molecular Biology, Faculty of Life Science, Mawlana Bhashani Science and Technology University, Tangail, 1902, Bangladesh. Email: mehedi.bmb.mbstu@gmail.com.

## Declaration of competing interest

NA.
